# Crystal structure and Hirshfeld surface analysis of 5-hy­droxy­penta­nehydrazide

**DOI:** 10.1107/S2056989024002871

**Published:** 2024-04-09

**Authors:** Guilherme Augusto Justen, José Severiano Carneiro Neto, Francielli Sousa Santana, Maria Leiriane Batistel Ribas, Felipe Gomes da Silva de Paula, Priscila Paola Dario, Marcelo Gonçalves Montes D’Oca

**Affiliations:** a Departamento de Química, Universidade Federal do Paraná, Centro, Politécnico, Jardim das Américas, 81530-900, Curitiba-PR, Brazil; Katholieke Universiteit Leuven, Belgium

**Keywords:** hydrazide, lactone, medicinal chemistry, crystal structure, 5-hy­droxypentanehydrazide

## Abstract

Carb­oxy­hydrazides are widely used in medicinal chemistry because of their medicinal properties and many drugs have been developed containing this functional group. A suitable inter­mediate to obtain potential hydrazide drug candidates is the title compound 5-hy­droxy­penta­nehydrazide, C_5_H_12_N_2_O_2_ (**1**). The aliphatic compound can react both *via* the hydroxyl and hydrazide moieties forming derivatives, which can inhibit Mycobacterium tuberculosis catalase-peroxidase (KatG) and consequently causes death of the pathogen. In this work, the hydrazide was obtained *via* a reaction of a lactone with hydrazine hydrate. The colourless prismatic single crystals belong to the ortho­rhom­bic space group *Pca*2_1_. Regarding supra­molecular inter­actions, the compound shows classic medium to strong inter­molecular hydrogen bonds involving the hydroxyl and hydrazide groups. Besides, the three-dimensional packing also shows weak H⋯H and C⋯H contacts, as investigated by Hirshfeld surface analysis (HS) and fingerprint plots (FP).

## Abstract 2

δ-Valerolactone was reacted with hydrazine hydrate in methanol affording crystals of 5-hy­droxy­penta­nehydrazide, which were characterized *via* single-crystal X-ray diffraction. The compound crystallizes in the ortho­rhom­bic space group *Pca*2_1_ without any crystallization solvent. The hydrazide functional group shows C—N and C=O bond lengths measuring 1.3376 (17) Å and 1.2375 (16) Å, respectively.

## 1. Chemical context

Carboxyhydrazides are non-alkaline compounds that can be identified as hydrazines containing an acyl group as one of their substituents, thus they are of general formula *R*_1_–N*R*_2_–N*R*_3_ *R*_4_, where *R*­_1_ is an acyl group and *R*_2_–*R*_4_ are typically hydrogen atoms or alkyl substituents. These compounds, particularly those in which *R*_2_–*R*_4_ are hydrogen atoms, present themselves as valuable functional groups for drug design, since compounds with this functional group and its derivatives tend to have biological activity as anti­depressants or anti­biotics, for example (Narang *et al.*, 2012 ▸). The medicinal potentiality led to the development of several drugs containing this functional group, such as isoniazid, furazolidone, and isocarboxazide (Gegia *et al.*, 2017 ▸). 
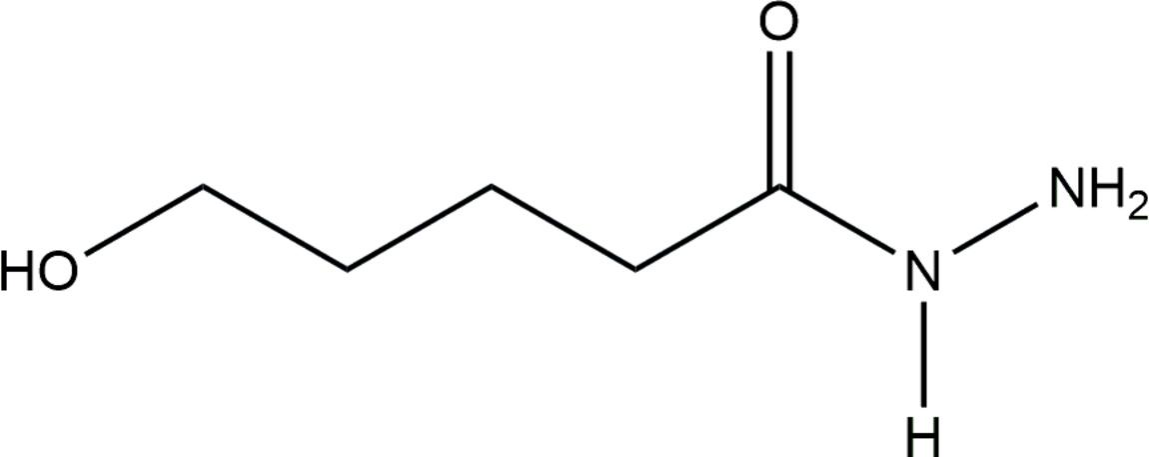


Hydrazides are usually formed *via* the reaction of hydrazine (usually obtained from its hydro­chloride or its hydrate) with acyl derivatives such as esters, acyl halides, or anhydrides (Huang *et al.*, 2016 ▸). For example, lactones (cyclic esters) promptly react with hydrazine hydrate in a polar solvent such as methanol. In this work, δ-valerolactone was added to hydrazine to afford ortho­rhom­bic crystals of 5-hy­droxy­penta­nehydrazide **1** (Huang *et al.*, 2016 ▸).

Compound **1** was first synthesized by Karakhanov and collaborators in 1969 (Karakhanov *et al.*, 1969 ▸). However, this is the first report describing the crystallographic features of 5-hy­droxy­penta­nehydrazide.

## 2. Structural commentary

**Figure 1:**
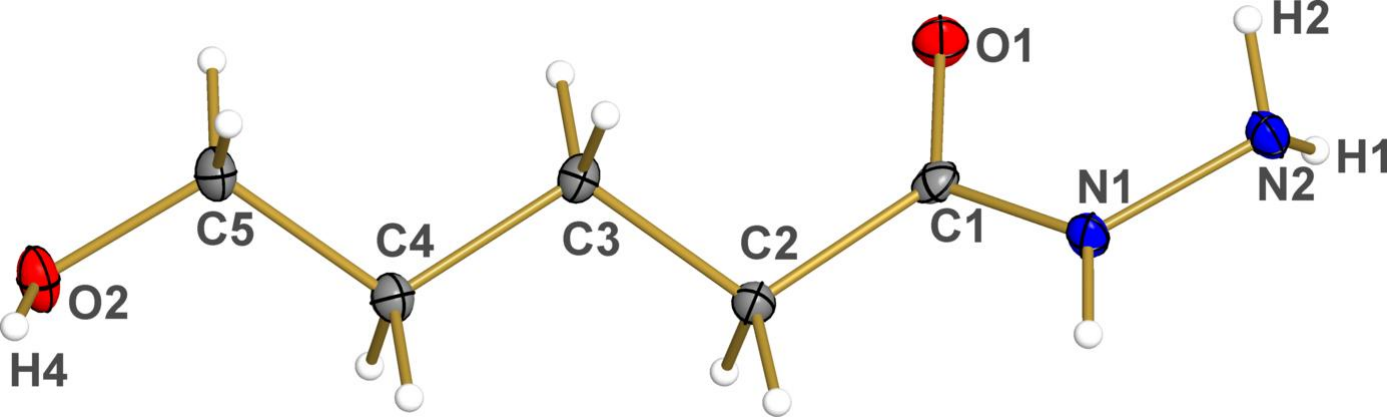
View of the mol­ecular structure of 5-hy­droxy­penta­nehydrazide (**1**) with the atom-numbering scheme. Displacement ellipsoids are drawn at the 50% probability level.

**Figure 2:**
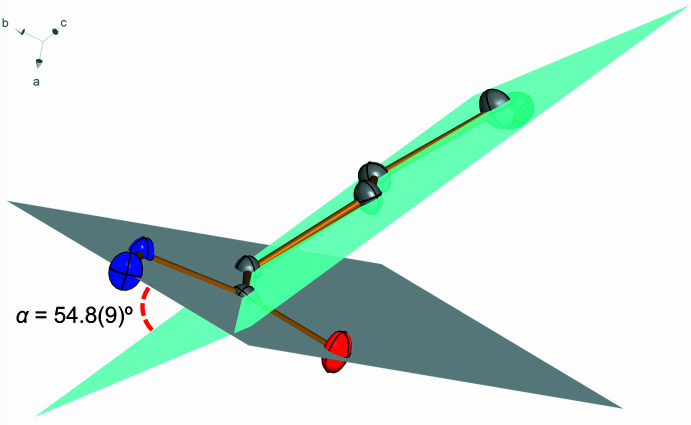
Representation of the dihedral angle *α* formed by the planes containing carbon chain atoms C1, C2, C3, C4, and C5 (blue plane) and the hydrazine group atoms N1, N2, O1, C1, and C2 (grey plane) in **1**. Grey: carbon; red: oxygen; blue: nitro­gen. H atoms atoms were omitted for clarity. Displacement ellipsoids are drawn at the 50% probability level.

The mol­ecule of 5-hy­droxy­penta­nehydrazide (Fig. 1 ▸), crystallizes in the ortho­rhom­bic space group *Pca*2_1_. The asymmetric unit comprises a unique mol­ecule of **1** with no atoms in special positions, as well as no solvent of crystallization. The C—N, C=O, and N—N bond lengths within the hydrazide group of 1.3376 (17) Å, 1.2375 (16) Å, and 1.4193 (14), respectively, are in agreement with the values reported for aliphatic compounds containing a hydrazide unit (Jensen, 1956 ▸; Lo *et al.*, 2020 ▸; Kolesnikova *et al.*, 2022 ▸). Moreover, the short and unbranched carbon chain formed by atoms C1, C2, C3, C4, and C5, is located in a plane (blue plane in Fig. 2 ▸) that makes an angle *α* of 54.8 (9)° relative to the plane containing the hydrazide atoms N1, N2, C1, C2 and O1 (grey plane in Fig. 2 ▸).

The aforementioned conformational features, linked to the orientation of the hydrazide group, are a relatively common characteristic in compounds containing these groups linked to carbon chains. The *α* angle of 54.8 (9)° observed in compound **1** is consistent with values reported in the literature, regardless of the carbon chain size. Noteworthy, values include 54.54° for a compound with a twelve-carbon chain (Jensen, 1956 ▸), 56.33° for a nine-carbon chain (Jensen & Lingafelter, 1961 ▸), and 57.08° for a six-carbon chain (Lee *et al.*, 2016 ▸).

## 3. Supra­molecular features

**Figure 3:**
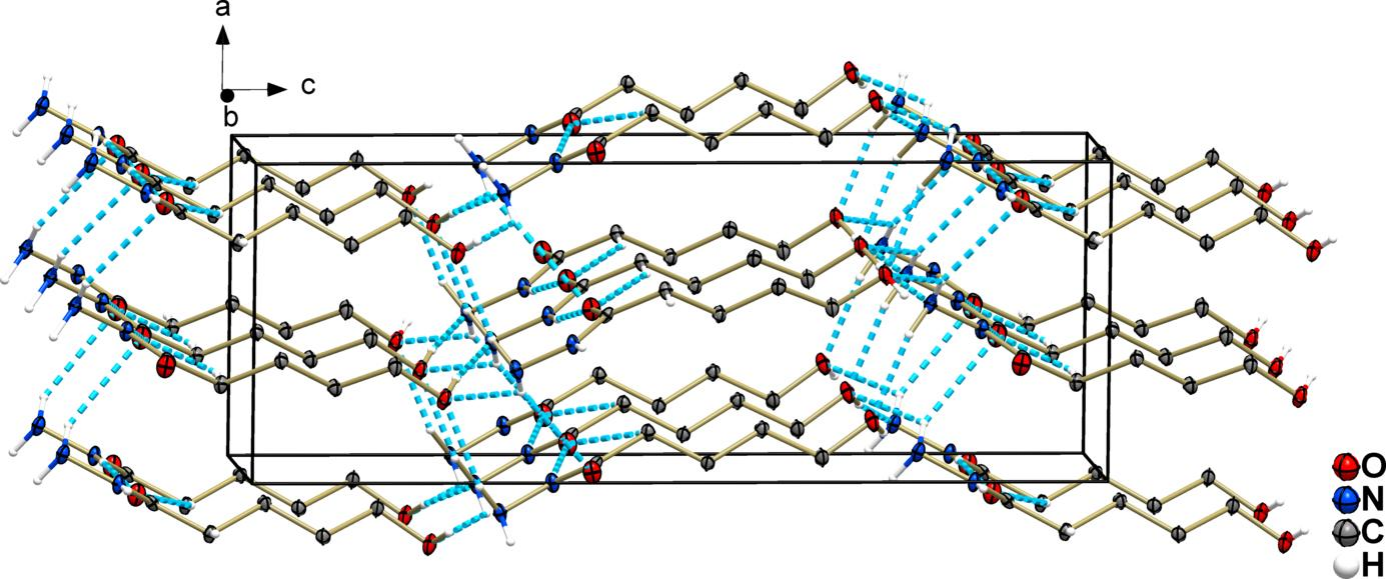
Crystal packing of **1** viewed along the *b* axis. Inter­molecular O—H⋯N, N—H⋯O and C—H⋯O hydrogen bonds are shown as dashed blue lines. H atoms not involved in hydrogen bonding were omitted for clarity.

**Figure 4:**
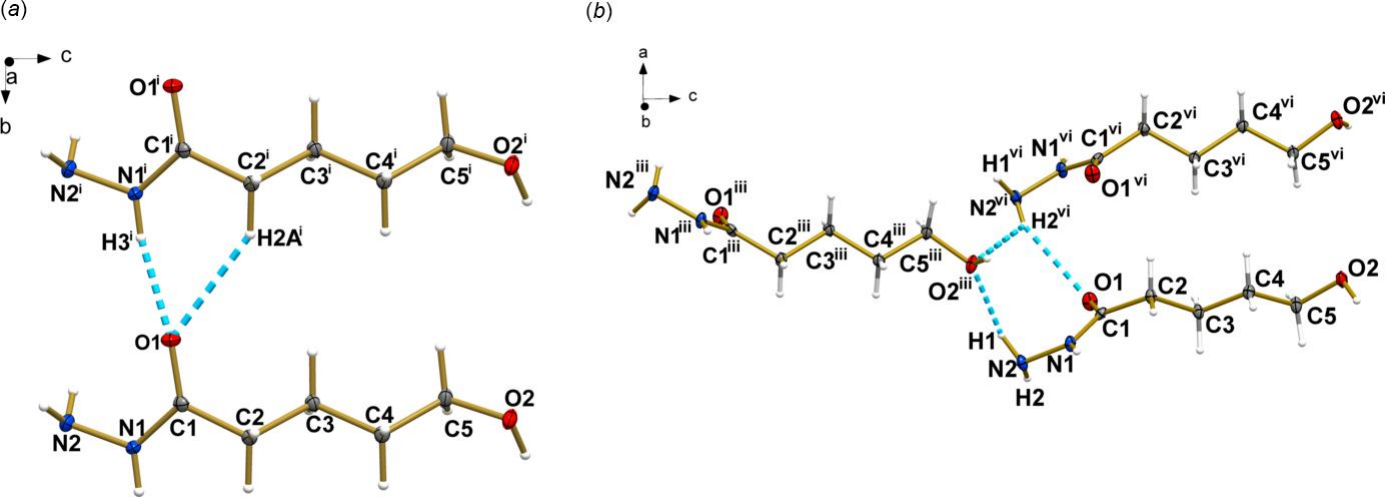
(*a*) Representation of the six-membered ring formed by N—H⋯O and C–H⋯O hydrogen bonds (blue dashed lines) between adjacent mol­ecules in the *b*-axis direction. (*b*) Representation of the seven-membered ring formed by N—H⋯O hydrogen bonds (blue dashed lines) between carbonyl and hydrazide groups of adjacent mol­ecules in the *a*-axis direction. Symmetry codes: (i) *x*, −*y* + 1, *z*; (iii) −*x* + 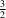 , *y*, *z* − 1; (vi) *x* + 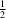 , −*y* + 1, *z*. Displacement ellipsoids are drawn at the 50% probability level.

The three-dimensional packing of **1** (Fig. 3 ▸) is characterized by several inter­molecular O–H⋯N and N–H⋯O hydrogen bonds involving the hydroxyl and hydrazine as hydrogen bond donor and acceptor groups (Table 1 ▸). Among them, the strongest ones, regarding shortest H⋯acceptor distances and most linear donor—H⋯acceptor angles, are the N1—H3⋯O1^ii^ [2.03 (2) Å; 166 (2)°; symmetry code: (ii) *x*, *y* + 1, *z*] and O2—H4⋯N2^i^ [1.92 (3) Å; 172 (2)°; symmetry code: (i) −*x* + 1, −*y* + 2, *z* + 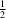 ], involving the hydrazine and hydroxyl as donor group, respectively, and carbonyl and hydrazine moieties as acceptor groups, respectively. Besides, the hydrazine moiety also promotes hydrogen bonds of medium-force: N2—H1⋯O2^iii^ [2.10 (2) Å; 147.2 (18)°; symmetry code: (iii) −*x* + 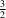 , *y*, *z* − 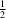 ], N2—H2⋯O1^iv^ [2.59 (2) Å; 144.0 (16)°; symmetry code: (iv) *x* − 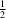 , −*y* + 1, *z*] and N2—H2⋯O2^v^ [2.57 (2) Å; 122.0 (16)°; symmetry code: (v) −*x* + 1, 1 − *y*, *z* − 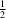 ]. The latter promotes the formation of chains with a *C*(9) graph-set motif running in the *c*-axis direction. A weak hydrogen bond of type C—H⋯O is also present in the crystal packing. Notably, thanks to this weak inter­action, the carbonyl group is the acceptor of a bifurcated hydrogen bond, sharing its electronic density with the C2—H2 and N2—H2 groups and resulting in a six-membered ring with an 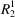 (6) graph-set motif in the *b*-axis direction (Fig. 4 ▸ *a*). The formation of seven-membered rings with an 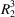 (7) graph-set motif is observed involving the H1–N2–N1–C1–O1⋯H2^vi^⋯O2^iii^ moiety in the *a*-axis direction [symmetry code: (iii) −*x* + 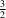 , *y*, *z* − 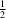 ; (vi) *x* + 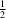 , −*y* + 1, *z*; Fig. 4 ▸ *b*). Together, these inter­actions act cooperatively for the stability of **1** in the solid state (Sutor *et al.*, 1962 ▸; Domagała & Grabowski, 2005 ▸).

The non-covalent inter­actions responsible for the crystal packing were also investigated by a Hirshfeld surface analysis (HS; Hirshfeld, 1977 ▸), performed with *CrystalExplorer 21.5* (Spackman *et al.*, 2021 ▸). The Hirshfeld surface provides a three-dimensional representation that elucidates mol­ecular inter­actions through the mathematical distance functions *d_i_*, denoting the distance from the surface to the nearest atom within it, and *d_e_*, denoting the distance from the surface to the nearest atom outside of it. The normalization of the *d*_i_ and *d*_e_ distances by the van der Waals radius leads to the *d_norm_* function, which enables the visualization of a surface that delineates regions involved in both accepting and donating inter­molecular inter­actions. A key component of this analysis entails the generation of 2D fingerprint plots (FP), providing two-dimensional representations of the Hirshfeld surface.

Using the *d_norm_* function, expressed by a colour scale, this method describes the strength of inter­atomic inter­actions. Red and blue indicate inter­atomic contacts where the distance between atoms is smaller or larger, respectively, than the sum of the van der Waals radii of the atoms involved, while white indicates contacts with distances close to the sum of the van der Waals radii.

**Figure 5:**
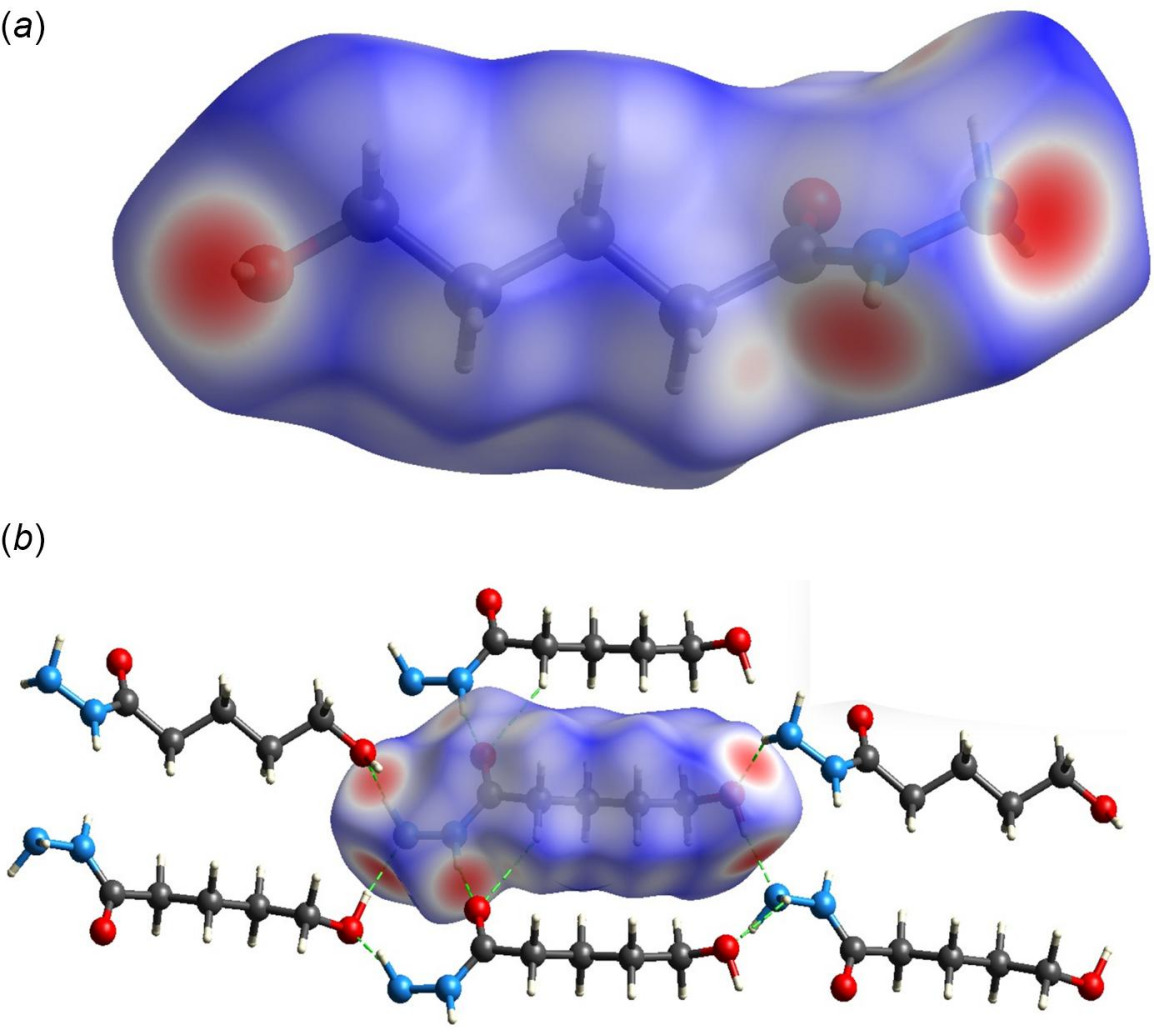
Representation of (*a*) the Hirshfeld surface for **1** plotted over *d_norm_* and (*b*) illustration of the N—H⋯O, O—H⋯N and C—H⋯O inter­actions depicted by dashed green lines.

In the case of compound **1**, the red colour in Fig. 5 ▸ *a* highlights the region of most intense contacts involving the nitro­gen, oxygen and hydrogen atoms from the hydrazide and hydroxyl groups with adjacent oxygen atoms. Fig. 5 ▸ *b* illustrates the nearest mol­ecules within the crystal packing, delineating the spatial arrangement of the shortest inter­actions. Meanwhile, blue surfaces, which indicate longer-range inter­actions, arise mainly from H⋯H contributions.

**Figure 6:**
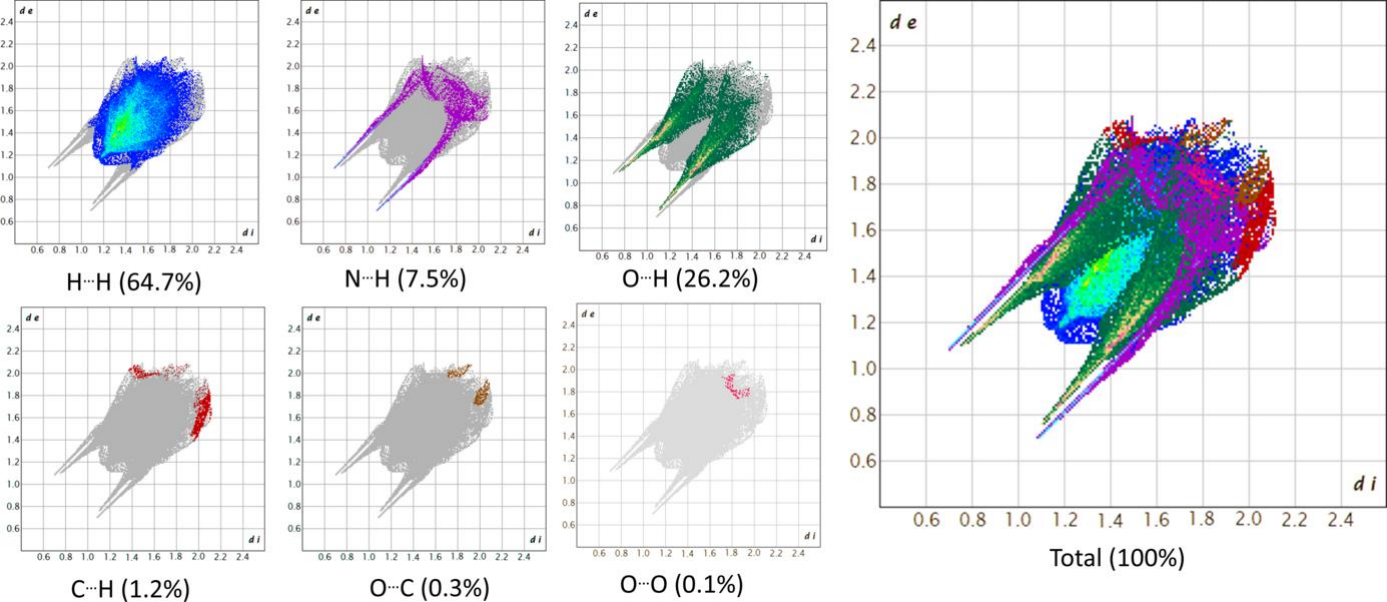
Fingerprint plots for **1** showing the total contribution of individual inter­actions and those delineated into H⋯H, N⋯H/H⋯N, O⋯H/H⋯O, C⋯H/H⋯C, O⋯C/C⋯O and O⋯O inter­actions.

Fingerprint plots (FP) were generated to qu­antify the contribution of each inter­atomic inter­action to the supra­molecular structure. For this purpose, the *d*_i_ (*x* axis) and *d*_e_ (*y* axis) distances, expressed in Ångstroms, of the HS are used. For **1**, the percentages of the surface area correspond to 64.7% for H⋯H, 26.2% for O⋯H/H⋯O, 7.5% for N⋯H/H⋯N, 1.2% for C⋯H/H⋯C, 0.3% for O⋯C/C⋯O and 0.1% for O⋯O inter­actions, as shown in Fig. 6 ▸.

## 4. Database survey

A survey of the Cambridge Structural Database (CSD2023.2.0, version 5.45, November 2023; Groom *et al.*, 2016 ▸) revealed several similar structures. 5-Hy­droxy­penta­nehydrazide was first synthesized as a byproduct of the reaction of di­hydro­pyran and phenyl azide (Karakhanov *et al.*, 1969 ▸) and has never had its structural properties discussed, although it was first obtained in its crystalline form. The synthesis and crystallographic characterization of other similar aliphatic hydrazide derivatives has been reported: *t*-butyl hydrazine­carboxyl­ate (CSD refcode RENZUJ; Aitken & Slawin, 2022 ▸), α-cyano­acetohydrazide (CYACHZ; Chieh, 1973 ▸), *n*-dodeca­noic acid hydrazide (DDEAHN; Jensen, 1956 ▸), hexa­nedihydrazide (MUYRIK; Lo *et al.*, 2020 ▸), *n*-nona­noic acid hydrazide (NONACH; Jensen & Lingafelter, 1961 ▸) and *n*-octa­noic acid hydrazide (ZZZOMM; Jensen & Lingafelter, 1953 ▸).

## 5. Synthesis and crystallization

To a round-bottom flask, δ-valerolactone (100 mg, 9.99 mmol), hydrazine hydrate (200 mg, 4.0 mmol) and 5 mL of methanol were added. The resulting solution was maintained stirring under reflux conditions for 24 h. The solution was then allowed to cool slowly to room temperature. After 20 minutes, a solid started to precipitate in the flask. The solid, which was filtered off and air dried, afforded 115.0 mg of colourless crystals of 5-hy­droxy­penta­nehydrazide (**1**) in 88.0% yield. The melting point (375–379 K) was in accordance with literature (Karakhanov *et al.*, 1969 ▸).

## 6. Refinement

Crystal data, data collection and structure refinement details are summarized in Table 2 ▸. The hydrogen atoms of the carbon chain were included in idealized positions with C—H distances set to 0.99 Å and refined using a riding model with *U*_iso_(H) = 1.2*U*_eq_(C); the other hydrogen atoms were located in difference-Fourier maps and were refined freely.

## Supplementary Material

Crystal structure: contains datablock(s) I. DOI: 10.1107/S2056989024002871/vm2296sup1.cif

Structure factors: contains datablock(s) I. DOI: 10.1107/S2056989024002871/vm2296Isup2.hkl

Supporting information file. DOI: 10.1107/S2056989024002871/vm2296Isup3.mol

Supporting information file. DOI: 10.1107/S2056989024002871/vm2296Isup4.cml

CCDC reference: 2345070

Additional supporting information:  crystallographic information; 3D view; checkCIF report

## Figures and Tables

**Table 1:** Hydrogen-bond geometry (Å, °)

*D*—H⋯*A*	*D*—H	H⋯*A*	*D*⋯*A*	*D*—H⋯*A*
O2—H4⋯N2^i^	0.87 (3)	1.92 (3)	2.7865 (15)	172 (2)
N1—H3⋯O1^ii^	0.85 (2)	2.03 (2)	2.8662 (14)	166 (2)
N2—H1⋯O2^iii^	0.91 (2)	2.10 (2)	2.9068 (16)	147.2 (18)
N2—H2⋯O1^iv^	0.82 (2)	2.59 (2)	3.2858 (16)	144.0 (16)
N2—H2⋯O2^v^	0.82 (2)	2.57 (2)	3.0789 (14)	122.0 (16)
C2—H2*A*⋯O1^ii^	0.99	2.57	3.4309 (15)	145

Symmetry codes: (i) 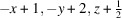 ; (ii) 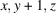 ; (iii) 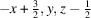 ; (iv) 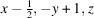 ; (v) 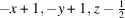 .

**Table 2:** Experimental details

Crystal data	
Chemical formula	C_5_H_12_N_2_O_2_
*M*_r_	132.17
Crystal system, space group	Orthorhombic, *P* *c* *a*2_1_
Temperature (K)	100
*a*, *b*, *c* (Å)	7.1686 (5), 4.8491 (3), 19.1276 (14)
*V* (Å^3^)	664.90 (8)
*Z*	4
Radiation type	Mo *K*α
μ (mm^−1^)	0.10
Crystal size (mm)	0.32 × 0.16 × 0.13

Data collection	
Diffractometer	Bruker D8 Venture/Photon 100 CMOS
Absorption correction	Multi-scan (*SADABS*; Krause *et al.*, 2015 ▸)
*T*_min_, *T*_max_	0.731, 0.746
No. of measured, independent and observed [*I* > 2σ(*I*)] reflections	26421, 1567, 1527
*R*_int_	0.029
(sin θ/λ)_max_ (Å^−1^)	0.655

Refinement	
*R*[*F* ^2^ > 2σ(*F* ^2^)], *wR*(*F* ^2^), *S*	0.022, 0.059, 1.08
No. of reflections	1567
No. of parameters	98
No. of restraints	1
H-atom treatment	H atoms treated by a mixture of independent and constrained refinement
Δρ_max_, Δρ_min_ (e Å^−3^)	0.21, −0.19
Absolute structure	Flack *x* determined using 732 quotients [(*I* ^+^)−(*I* ^−^)]/[(*I* ^+^)+(*I* ^−^)] (Parsons *et al.*, 2013 ▸)
Absolute structure parameter	−0.1 (2)

Computer programs: *APEX4* (Bruker, 2022 ▸), *SAINT* (Bruker, 2019 ▸), *SHELXT2015* (Sheldrick, 2015*a* ▸), *SHELXL2019/2* (Sheldrick, 2015*b* ▸), *DIAMOND* (Brandenburg & Putz, 1999 ▸) and *WinGX* (Farrugia, 2012 ▸).

## supplementary crystallographic information

### Crystal data

**Table:**

C_5_H_12_N_2_O_2_	*D*_x_ = 1.320 Mg m^−^^3^
*M**_r_* = 132.17	Mo *K*α radiation, λ = 0.71073 Å
Orthorhombic, *P**c**a*2_1_	Cell parameters from 9932 reflections
*a* = 7.1686 (5) Å	θ = 4.2–28.7°
*b* = 4.8491 (3) Å	µ = 0.10 mm^−^^1^
*c* = 19.1276 (14) Å	*T* = 100 K
*V* = 664.90 (8) Å^3^	Prism, colourless
*Z* = 4	0.32 × 0.16 × 0.13 mm
*F*(000) = 288	

### Data collection

**Table:**

Bruker D8 Venture/Photon 100 CMOS diffractometer	1567 independent reflections
Radiation source: fine-focus sealed tube	1527 reflections with *I* > 2σ(*I*)
Detector resolution: 10.4167 pixels mm^-1^	*R*_int_ = 0.029
φ and ω scans	θ_max_ = 27.8°, θ_min_ = 4.3°
Absorption correction: multi-scan (SADABS; Krause *et al.*, 2015)	*h* = −9→9
*T*_min_ = 0.731, *T*_max_ = 0.746	*k* = −6→6
26421 measured reflections	*l* = −25→25

### Refinement

**Table:**

Refinement on *F*^2^	Secondary atom site location: difference Fourier map
Least-squares matrix: full	Hydrogen site location: mixed
*R*[*F*^2^ > 2σ(*F*^2^)] = 0.022	H atoms treated by a mixture of independent and constrained refinement
*wR*(*F*^2^) = 0.059	*w* = 1/[σ^2^(*F*_o_^2^) + (0.0379*P*)^2^ + 0.0769*P*] where *P* = (*F*_o_^2^ + 2*F*_c_^2^)/3
*S* = 1.08	(Δ/σ)_max_ < 0.001
1567 reflections	Δρ_max_ = 0.21 e Å^−^^3^
98 parameters	Δρ_min_ = −0.19 e Å^−^^3^
1 restraint	Absolute structure: Flack *x* determined using 732 quotients [(*I*^+^)-(*I*^-^)]/[(*I*^+^)+(*I*^-^)] (Parsons *et al.*, 2013)
Primary atom site location: dual	Absolute structure parameter: −0.1 (2)

### Special details

**Table:**

Geometry. All esds (except the esd in the dihedral angle between two l.s. planes) are estimated using the full covariance matrix. The cell esds are taken into account individually in the estimation of esds in distances, angles and torsion angles; correlations between esds in cell parameters are only used when they are defined by crystal symmetry. An approximate (isotropic) treatment of cell esds is used for estimating esds involving l.s. planes.

### Fractional atomic coordinates and isotropic or equivalent isotropic displacement parameters (Å^2^)

**Table:**

	*x*	*y*	*z*	*U*_iso_*/*U*_eq_	
O1	0.58285 (13)	0.44491 (18)	0.38116 (6)	0.0149 (2)	
O2	0.71437 (14)	0.7429 (2)	0.71584 (5)	0.0151 (2)	
N1	0.51145 (15)	0.8787 (2)	0.34743 (5)	0.0105 (2)	
N2	0.42516 (17)	0.7936 (2)	0.28423 (6)	0.0122 (2)	
C1	0.58629 (18)	0.6966 (3)	0.39182 (6)	0.0097 (2)	
C3	0.58864 (17)	0.6955 (2)	0.52353 (6)	0.0110 (2)	
H3A	0.453999	0.740443	0.525239	0.013*	
H3B	0.601004	0.492251	0.522213	0.013*	
C4	0.68313 (18)	0.8053 (3)	0.58941 (6)	0.0115 (3)	
H4A	0.818747	0.768536	0.586516	0.014*	
H4B	0.665552	1.007624	0.591765	0.014*	
C5	0.60650 (19)	0.6753 (3)	0.65582 (7)	0.0139 (3)	
H5A	0.476648	0.738769	0.663034	0.017*	
H5B	0.603920	0.472447	0.650158	0.017*	
C2	0.67320 (18)	0.8176 (2)	0.45691 (6)	0.0106 (2)	
H2A	0.654542	1.019919	0.456926	0.013*	
H2B	0.809132	0.781623	0.456417	0.013*	
H1	0.514 (3)	0.715 (4)	0.2573 (12)	0.024 (5)*	
H2	0.345 (3)	0.680 (4)	0.2935 (10)	0.016 (4)*	
H3	0.517 (3)	1.052 (4)	0.3531 (11)	0.020 (5)*	
H4	0.664 (4)	0.890 (5)	0.7337 (13)	0.034 (6)*	

### Atomic displacement parameters (Å^2^)

**Table:**

	*U*^11^	*U*^22^	*U*^33^	*U*^12^	*U*^13^	*U*^23^
O1	0.0227 (5)	0.0074 (4)	0.0146 (4)	0.0001 (3)	−0.0016 (4)	−0.0006 (3)
O2	0.0204 (5)	0.0158 (4)	0.0092 (4)	0.0034 (4)	−0.0026 (4)	−0.0033 (4)
N1	0.0161 (5)	0.0070 (5)	0.0084 (5)	−0.0001 (4)	−0.0012 (4)	−0.0011 (4)
N2	0.0160 (5)	0.0111 (5)	0.0094 (5)	−0.0012 (4)	−0.0029 (4)	−0.0016 (4)
C1	0.0106 (5)	0.0096 (5)	0.0090 (6)	−0.0006 (4)	0.0033 (4)	0.0005 (4)
C3	0.0127 (5)	0.0120 (5)	0.0082 (5)	−0.0013 (4)	0.0005 (4)	0.0012 (5)
C4	0.0132 (6)	0.0120 (6)	0.0092 (5)	−0.0010 (4)	0.0003 (5)	−0.0009 (5)
C5	0.0170 (6)	0.0166 (6)	0.0081 (5)	−0.0031 (5)	−0.0008 (5)	−0.0007 (5)
C2	0.0136 (6)	0.0093 (5)	0.0088 (5)	−0.0014 (5)	−0.0004 (5)	0.0002 (4)

### Geometric parameters (Å, º)

**Table:**

O1—C1	1.2375 (16)	C3—C2	1.5303 (16)
O2—C5	1.4223 (16)	C3—H3A	0.9900
O2—H4	0.87 (3)	C3—H3B	0.9900
N1—C1	1.3376 (17)	C4—C5	1.5208 (17)
N1—N2	1.4193 (14)	C4—H4A	0.9900
N1—H3	0.85 (2)	C4—H4B	0.9900
N2—H1	0.91 (2)	C5—H5A	0.9900
N2—H2	0.82 (2)	C5—H5B	0.9900
C1—C2	1.5109 (16)	C2—H2A	0.9900
C3—C4	1.5265 (16)	C2—H2B	0.9900
			
C5—O2—H4	106.4 (17)	C5—C4—H4A	109.1
C1—N1—N2	121.55 (11)	C3—C4—H4A	109.1
C1—N1—H3	123.8 (15)	C5—C4—H4B	109.1
N2—N1—H3	114.6 (15)	C3—C4—H4B	109.1
N1—N2—H1	107.3 (14)	H4A—C4—H4B	107.8
N1—N2—H2	108.6 (13)	O2—C5—C4	112.48 (10)
H1—N2—H2	109.6 (19)	O2—C5—H5A	109.1
O1—C1—N1	122.60 (12)	C4—C5—H5A	109.1
O1—C1—C2	121.81 (11)	O2—C5—H5B	109.1
N1—C1—C2	115.58 (11)	C4—C5—H5B	109.1
C4—C3—C2	112.13 (10)	H5A—C5—H5B	107.8
C4—C3—H3A	109.2	C1—C2—C3	111.87 (10)
C2—C3—H3A	109.2	C1—C2—H2A	109.2
C4—C3—H3B	109.2	C3—C2—H2A	109.2
C2—C3—H3B	109.2	C1—C2—H2B	109.2
H3A—C3—H3B	107.9	C3—C2—H2B	109.2
C5—C4—C3	112.62 (10)	H2A—C2—H2B	107.9
			
N2—N1—C1—O1	−0.98 (19)	O1—C1—C2—C3	−55.33 (16)
N2—N1—C1—C2	180.00 (11)	N1—C1—C2—C3	123.70 (11)
C2—C3—C4—C5	−177.29 (11)	C4—C3—C2—C1	176.88 (10)
C3—C4—C5—O2	170.30 (10)		

### Hydrogen-bond geometry (Å, º)

**Table:**

*D*—H···*A*	*D*—H	H···*A*	*D*···*A*	*D*—H···*A*
O2—H4···N2^i^	0.87 (3)	1.92 (3)	2.7865 (15)	172 (2)
N1—H3···O1^ii^	0.85 (2)	2.03 (2)	2.8662 (14)	166 (2)
N2—H1···O2^iii^	0.91 (2)	2.10 (2)	2.9068 (16)	147.2 (18)
N2—H2···O1^iv^	0.82 (2)	2.59 (2)	3.2858 (16)	144.0 (16)
N2—H2···O2^v^	0.82 (2)	2.57 (2)	3.0789 (14)	122.0 (16)
C2—H2*A*···O1^ii^	0.99	2.57	3.4309 (15)	145

Symmetry codes: (i) −*x*+1, −*y*+2, *z*+1/2; (ii) *x*, *y*+1, *z*; (iii) −*x*+3/2, *y*, *z*−1/2; (iv) *x*−1/2, −*y*+1, *z*; (v) −*x*+1, −*y*+1, *z*−1/2.
